# Solitonic State in Microscopic Dynamic Failures

**DOI:** 10.1038/s41598-018-38037-w

**Published:** 2019-02-13

**Authors:** H. O. Ghaffari, W. A. Griffith, M. Pec

**Affiliations:** 1 Department of Earth, Atmospheric and Planetary Sciences, Massachusetts Institute of Technology, Cambridge, Massachusetts USA; 2 School of Earth Sciences, Ohio State University, Columbus, Ohio USA

## Abstract

Onset of permanent deformation in crystalline materials under a sharp indenter tip is accompanied by nucleation and propagation of defects. By measuring the spatio-temporal strain field near the indenter tip during indentation tests, we demonstrate that the dynamic strain history at the moment of a displacement burst carries characteristics of the formation and interaction of local excitations, or solitons. We show that dynamic propagation of multiple solitons is followed by a short time interval where the propagating fronts can accelerate suddenly. As a result of such abrupt local accelerations, duration of the fast-slip phase of a failure event is shortened. Our results show that formation and annihilation of solitons mediate the microscopic fast weakening phase, during which extreme acceleration and collision of solitons lead to non-Newtonian behavior and Lorentz contraction, i.e., shortening of solitons’ characteristic length. The results open new horizons for understanding dynamic material response during failure and, more generally, complexity of earthquake sources.

## Introduction

Displacement bursts, apparent discontinuities in load-penetration depth (*P*-*h*) curves from indentation tests^1–4^, are usually accompanied by emission of ultrasound waves as well as charged particles and electrons^5–7^. Numerical studies and post-mortem observations have established that such excitations are the result of the nucleation of defects, which are the carriers of plasticity and are expressed as displacement bursts in *P-h* curves^2–4^. A burst represents single or multiple slip events which relax accumulated stresses under the indenter and perturb the strain field. Recent experimental studies have shown that the general process of splitting matter – in both gas and solid states – evolves over at least two main time scales due to distinct fast and slow relaxation processes^8–11^. The fast relaxation is manifested as an abrupt decrease in load in a displacement rate-controlled experiment or as a displacement burst in a load-controlled experiment. Hereafter we refer to the fast relaxation phase as the *weakening phase*. The weakening phase is usually followed by asymptotic relaxation to a new equilibrium state^8,9,11^. However, the dynamic role of nucleated defects in shaping the relaxation path and the mechanisms by which contact-induced plasticity leads to sharp decreases in load following a displacement bursts is still poorly understood. Here, we infer the relative change of a dynamic spatio-temporal strain field in the vicinity of the indenter tip at the onset of displacement bursts from recorded ultrasound waves. Our results show the existence of local nonlinear excitations hidden in emitted ultrasound waveforms. These solitons are revealed in network representations of the waveforms. In particular, we uncover a peculiar mechanism in which multiple solitons propagate at different velocities but converge to collide and annihilate each other during a characteristic Λ-shaped collision. Such convergence occurs on a very short time scale and leads to fast acceleration of solitons. We show that the latter solitons clearly squeeze their characteristic length, satisfying Lorentz’ contraction law.

## Results

**Figure 1:**
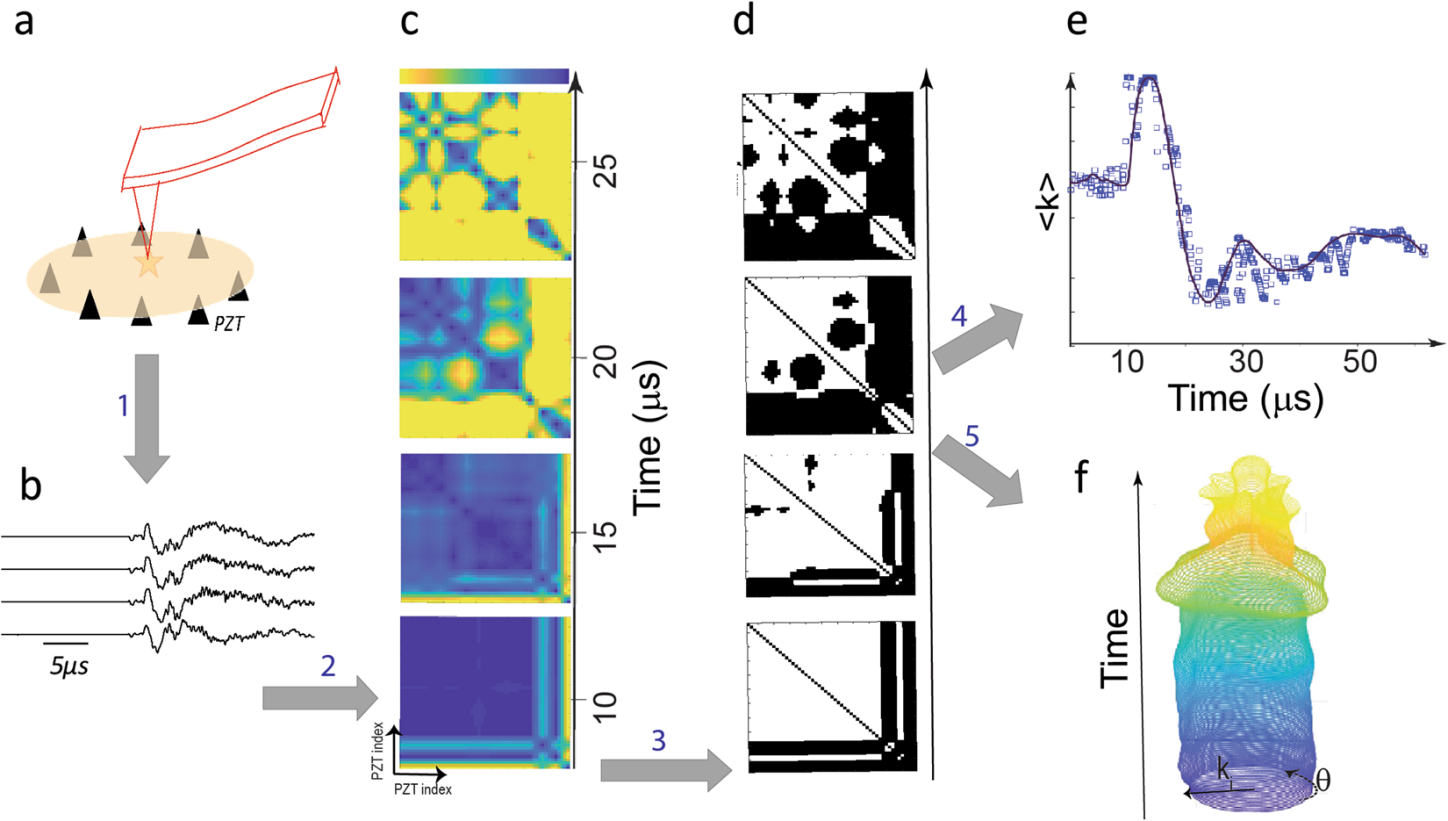
Summary of the employed method to construct k-chains from recorded (PZTs) passive ultrasound emissions. (**a**) Indentation of a film of a mineral suspended on piezoelectric elements-the source is located in the center of the film. **(b)** The recorded waveforms due to single acoustic emission event. **(c)** Equal-time similarity matrix in 4 successive snapshots. The most similar site-pairs are colored in blue. **(d)** The thresholded similarity matrix (i.e., adjacency matrix) in the shown snapshots. **(e)** Average of the most similar sites (most correlated nodes) as <k> -parameter indicates an approximation to relaxation path of the system. Calibration of <k(t)> shows that this parameter can be approximated with the evolution of mean strain field (see Figs S3 and S4). **(f)** To visualize evolution of the system –i.e., artificial lattice in 1D with periodicity - we consider a ring with components of nodes and assigning a radius at each site proportional with the number of links (*k*_*i*_). With this procedure, we map the ring-structure of the PZTs into a quasi-1D (dynamic) lattice where we derive kinetic energy of the lattice as well as quasimomentum-energy space of the lattice.

**Figure 2:**
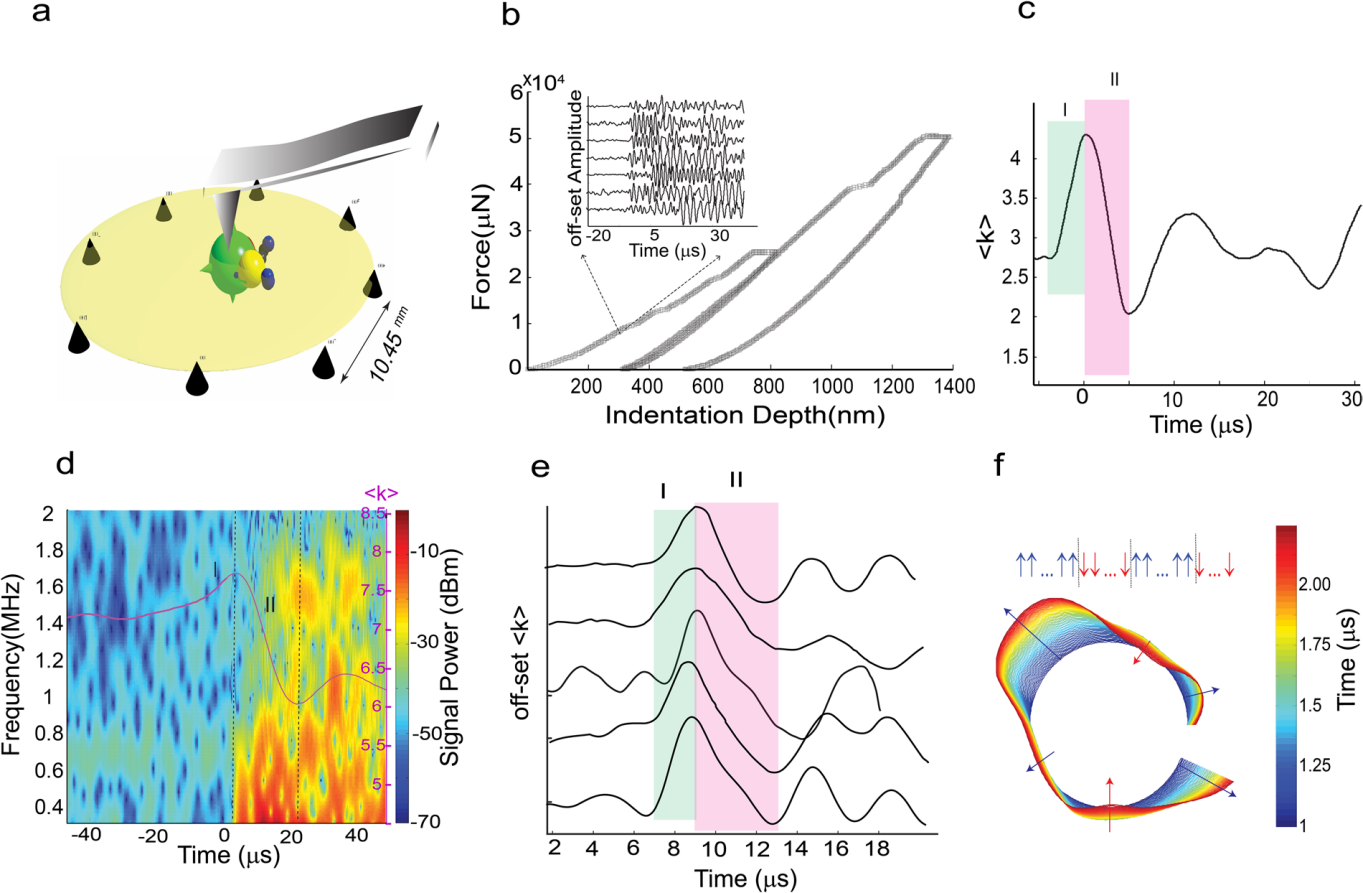
Displacement bursts and ultrasound emissions in indentation of suspended Mica-films. (**a**) Schematic representation of indentation. Thin Mica sheet is suspended over 8 piezoelectric transducers. The spheres correspond to locations where acoustic phonons are emitted as determined by a source location algorithm. The mica film covers all sensors and slightly exceeds the source-receiver distance. (**b**) Two loading-unloading paths with clear bursts events accompanied by emission of ultrasound waves. Inset shows an array of recorded emissions during the loading stage. (**c**) Evolution of the average of all nodes’ degree <k> for waveforms shown in (**b**) showing two main stages of relaxation. (**d**) High frequency components of an excited signal in our indentation test are mapped onto fast-weakening phase in <k(t)> onset of the fast-slip coincides with the broadening of the power spectrum. An overlapped (80%) 2,048-point fast Fourier transform is used to calculate the power spectral density. In (**e**) we show 5 different acoustic events transformed to <k(t)>, where <k(t)> represents time-evolution of the mean dynamic strain field over the spatially distributed sites. After initial rising phase (Phase I), a fast relaxation phase as the sharp drop of strain (phase II) is followed. Further relaxation occurs on longer timescales. See Fig. S1 for full waveforms of the shown k-profiles. (**f**) Accumulated k-chain patterns in ≈1.25 µs time-interval in transition from phase I to phase II for the plot shown in (**c**). The plot is the snapshot in vicinity of the peak of <k>; blue and red arrows show the trend of evolution or polarization of the chain in terms of the positive (growth-↑) or negative (folding-↓) rates respectively. Also see Supplementary Movie 1 as a 3d visualization of accumulated k-chains.

We indented suspended thin sheets of single crystal mica and recorded the emitted ultrasound signals (high frequency analogues to seismic waves)^12,13^ using an array of 8 to 16 ultrasound sensors arranged in a ring-like pattern around the indentation site (Fig. 1a and S3- see Methods section). The displacement bursts – coinciding with emission of high frequency noises – in our load-controlled experiment appear as discontinuities in the displacement record^1–4^. During an indentation test of the mica sheet, we were able to record tens of ultrasound excitations well known as acoustic emissions (AEs) with different emission levels of source energy. In Fig. 2b, we show waves from a single acoustic emission event parallel to the [001] direction of a muscovite mica specimen. Upon occurrence of an AE, the emitted waves carry information regarding the local disturbances in the vicinity of indenter tip, and evolution of such disturbances are reflected in the radiated waves.

To analyze the array of recorded AE signals, we use a thresholded similarity measure between sensor activities (measured in *mV*) and construct a virtual “lattice” of nodes that evolve throughout the recorded time series based on the behavior of the entire system. The main steps to construct a virtual (1D) lattice structure from an array of records are as follows (see Methods section for details -Fig. 1): (1) the waveforms recorded at each piezoelectric sensor are normalized to the maximum value of the amplitude at that site. (2) At each time step or generally in an interval of time, the amplitude of the *j*^th^ time-step from the *i*^th^ site ($1\le i\le N$) is denoted by ${u}^{i,j}(t)$ (in units of *mV*). *N* is the number of sites or acoustic sensors (3) ${u}^{i,j}(t)$ is compared with ${u}^{k,j}(t)$ to create links between the nodes. This means if $d({u}^{i,j}(t),\,{u}^{k,j}(t))\le \zeta$ (where ζ is the threshold level) we set ${a}_{ik}(j)=1$ otherwise ${a}_{ik}(j)=0$ where ${a}_{ik}(j)$ is the component of the connectivity matrix and $d(\,\cdot \,)=|{u}^{i,j}(t)-{u}^{k,j}(t)|$ is the employed *similarity metric* (Fig. 1c). This establishes equal-time similarity between sites. The result of this algorithm is an adjacency matrix (Fig. 1d) with components given by $a({x}_{i}(t),\,{x}_{k}(t))={\rm{\Theta }}(\zeta -|{u}^{i,j}(t)-{u}^{k,j}(t)|)$. Here ${\rm{\Theta }}(\,\cdot \,)$ is the Heaviside function.

The constructed one-dimensional artificial active lattice is characterized by the average of all nodes’ degree (<k>-or mean coordinate number) and evolution of the lattice topology allows us to study the complexity of the AE sources (Fig. 2c,d). The degree *k*_i_ of the *i*^th^ node at a given time represents the number of connected links to the node (Fig. 1e) where “links” are established based on a similarity metric, therefore representing the intensity of spatial correlation between the node and all other nodes^13–18^. Calibration of *k(t)* with impulsive compressive and shear sources indicates that the evolution of this parameter can be closely approximated as the relative change of dynamic strain (Methods section -Figs S3 and S4).

After establishing the intensity of equal-time similarity between sites and to visualize the temporal evolution of the dynamics of sites, we map the spatial evolution of the degree *k*_*i*_ of the *i*^th^ node, using polar coordinates ${({R}_{i},{\theta }_{i})}_{i=1,\mathrm{...},Nodes}$ where ${R}_{i}={k}_{i}$ and *θ*_*i*_ indicate the position of the node around the ring (we call this structure a *k-chain*-Fig. 2e,f- Supplementary Movie 1). Considering the periodicity of our geometry, the latter mapping assigns an intensity parameter to each site along a circle. This helps us to investigate the properties of k-chains in pseudo-momentum space, where a 1D-perioidc geometry is equivalent to the 1D Brillouin zone^19^. We will elaborate on this manifestation of k-chains when we extract 1D energy band structures of our dynamic lattice. Furthermore, it is convenient for our purposes to assign a polarization state for each node by ${s}_{i}=sign(\frac{\partial {k}_{i}}{\partial t})$ so that ${s}_{i}=\pm 1$ -see Fig. 2f.

Evaluating the spatio-temporal pattern of k_i_(t) as relative change of the strain field and its spatial mean trend <k(t)>, we found distinct patterns of dynamic failure characterized by (Fig. 2e): (I) First, <k> increases over a short time period (1–3 µs), followed by (II) a fast-slip phase (≈3–15 µs). Comparison of the Fourier transform of the recorded waveforms with <k(t)> -Fig. 2d- shows that the onset of high frequency components coincide with the fast-weakening phase in <k(t)> indicating sweeping from low energy (phase I) to high energy (phase II) level (Fig. S5). In phase I, the system is pulled from an equilibrium state to a state where all nodes polarize in the outward direction. This occurs in the rising part of <k(t)> in Fig. 2c and is stable for ~200–500 ns. After this short stable phase, the trend of correlation between nodes breaks down and one or more nodes’ states reverse; the reversed node forms a kink or domain wall in the strain field and represents a perturbation to the fully correlated state which interpolate between two fully correlated-states of the system-Fig. S7. The formation of kinks is visualized by the onset of “folding” of the k-chain, and we use the general term “soliton” to describe moving kinks (Fig. 2f- supplementary movie 1). Solitons are non-periodic waves that can be well-described by step-like functions of the form *tanh(θ-v*_*soliton*_*t/w)* where *v*_soliton_ is the propagation velocity of a soliton with a width, *w*, and position, *θ*^20,21^. From a physical point of view, a kink can be viewed as a local defect, and such defects – as we will show in detail below – are closely related to the dynamic strain history during failure. Here we define failure as the rapid drop of <k> as the indicator of the mean strain. The relevance of kinks for deformation can be tracked down to the pioneering work by Frenkel on the shear strength of crystalline solids and later the corresponding well-studied one-dimensional Frenkel-Kontorova (FK) model^21,22^, describing a 1D chain of finite coupled sites subjected to a sinusoidal potential. Processes pertaining resistance against deformation in the FK model are governed by excitation of kinks^22^.

**Figure 3:**
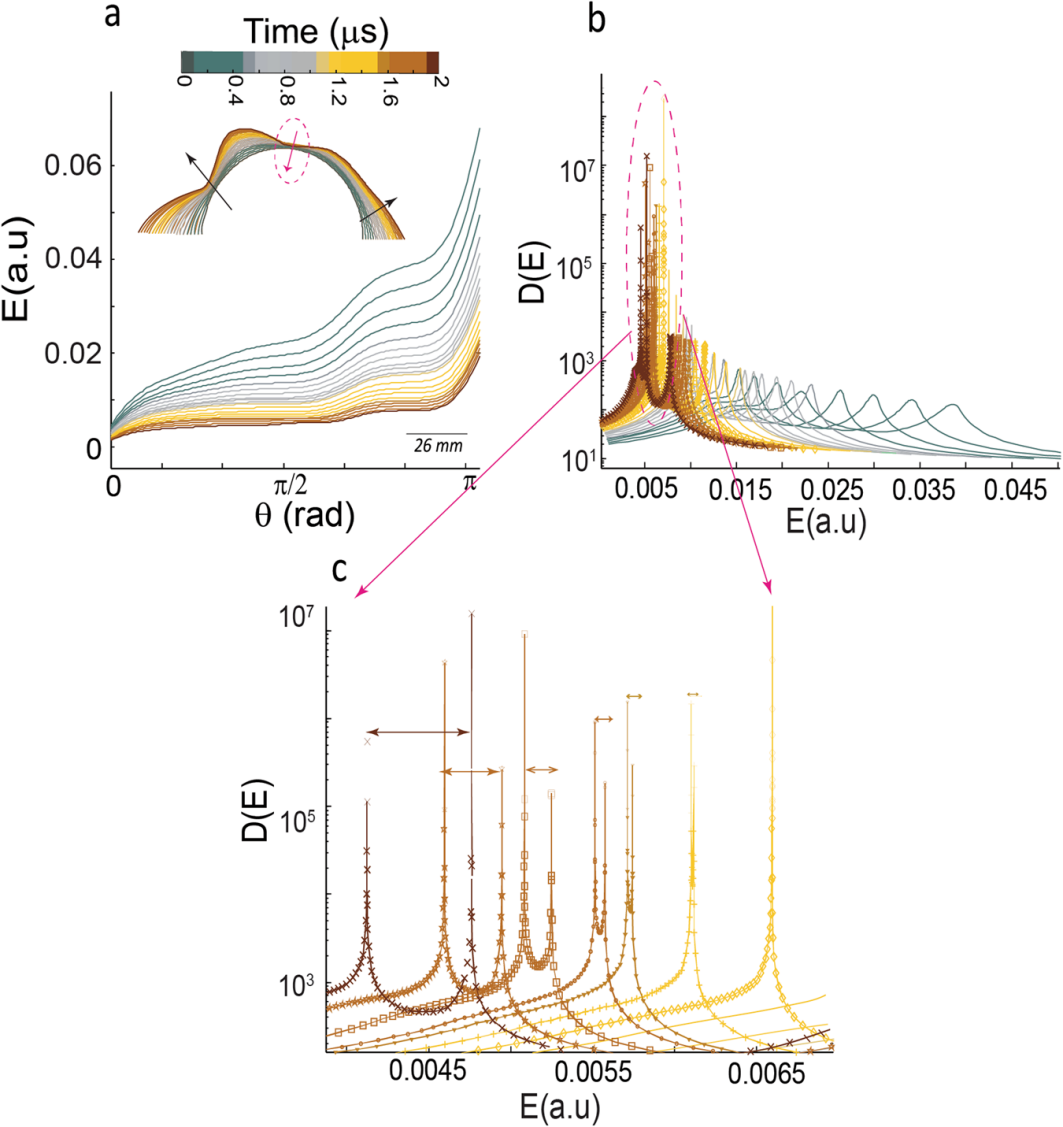
Formation and propagation of soliton-pairs. (**a**) The energy spectrum, E (in arbitrary units) as a function of direction Ө in different time steps. The kinetic energy of the chain decreases upon approaching nucleation of soliton shown by red arrow at the onset of folding of the k-chain. (**b**) Singularity in density profiles coincides with nucleation of a soliton pair. (**c**) Shows the zoom-out of onset and then splitting Van Hove singularities, which is manifested in propagation of soliton-pairs. The arrows show the separation of the solitons. Approaching E → 0 in (**b**) indicates that the moving kinks are the dominant state of the deformation of the chain.

To further illuminate the evolution of moving solitons in our chain, including their interactions and resultant effects on the inferred dynamic strain field, we present the evolution of density profiles based on kinetic energy of the k-chain: $E=\frac{1}{2}\,{\sum _{i=1}^{N}{m}_{i}(\frac{d{u}_{i}}{dt})}^{2}$ where $\frac{d{k}_{i}}{dt}\equiv \frac{d{u}_{i}}{dt}$ as the rate of normal displacement (i.e., dilatational components), N is the number of sites, and we assume a non-dimensional mass *m*_*i*_ = 1. The above kinetic energy term only considers the radial deformation of the chain (i.e., expansion or contraction) and energies corresponding to angular deformation has been omitted. We map the angular direction along a k-chain on to a vector **q** with fixed magnitude and direction *Ө*. Therefore, a system with *N* elements is characterized by **q** vectors and the energy *E* is defined in **q**-space (*E*(**q**)). To characterize the energy distribution in **q**-space (pseudo-momentum space), we use the concept of density of states in a 1-d chain as^19,20^: $D(E)\equiv \frac{1}{2\pi |\frac{dE}{d\theta }|}$ where $\theta =\frac{n\pi }{Na}$ and *n* = 1, …, *N* (*a* is the lattice space). If $\frac{dE}{d\theta }\to 0$, *D*(*E*) will include singularities known as *Van Hove singularities-VHS*^23,24^. Figure 3a shows an example of time-evolution of a k-chain in $0\le \theta \le \pi$ at the onset of a soliton-antisoliton pair formation. Approaching nucleation of the soliton, the kinetic energy of the chain monotonically decreases and apparently is absorbed at certain points which are the points of soliton nucleation. In fact, the kinetic energy includes additional term regarding non-radial motion (i.e., shear deformation) due to motion of kinks. Interestingly, the nucleation points correspond to points with divergences in the density of states (VHS) and splitting of VHS (Fig. 3c) coincides with soliton-antisoliton propagation.

**Figure 4:**
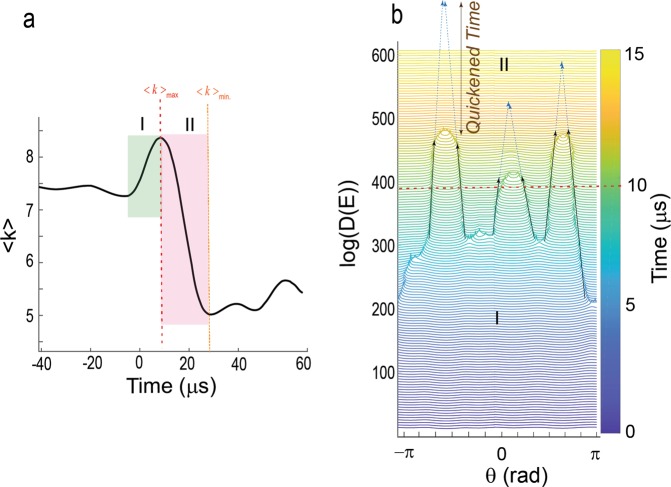
Accelerated moving defects quicken the fast-slip phase of dynamic failures. (**a**) An example of time-evolution of a k-chain as encoded in <k(t)> proportional with mean strain field. **(b)** Density profiles obtained between 0 µs to 15 µs in 0.1 µs increments are shifted vertically for clarity. We show the hypothetical trajectories of moving kinks (dotted arrows) without acceleration which accumulatively could increase the observed duration of the weakening phase by ~12 µs compared to the duration of the weakening phase with the accelerated fronts. Therefore, curved collision of solitons results earlier transition to the weakening phase (phase II). Such earlier transition yields a possible quickening mechanism in I → II. Also see Supplementary Movie 2.

**Figure 5:**
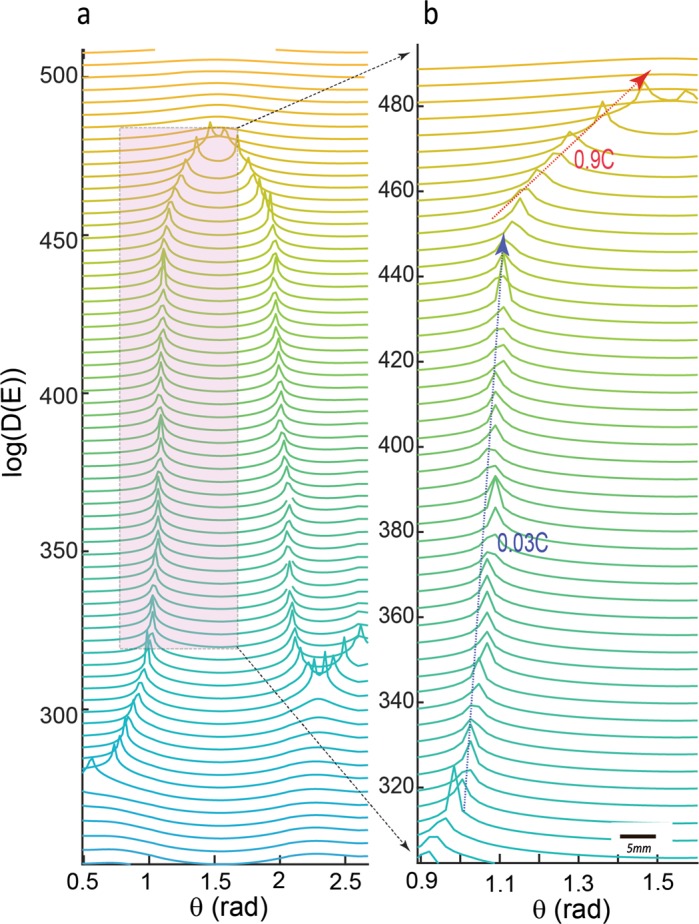
(**a**,**b**) Λ-shaped collision of two solitons. In this example, the velocity of a soliton accelerates from 0.03C to 0.9C m/s. The Λ-shaped collision is a universal feature of phase I → II transition. A close inspection of collisions indicates a parabolic behavior of fronts, i.e., a deviation from the linear trend. The color-code is similar to Fig. 4b.

In Figs 4 and 5, we show the time-evolution of *D(E)* in *θ-t* parameter space where several solitons nucleate, propagate and finally annihilate. A soliton moves with a certain velocity *v*_*soliton*_ and perturbs the initial state of the system (Figs 4 and S7). In Fig. 4, we show the nucleation, propagation and interaction of solitons which occurs in the transition from phase I to phase II. During this transition, we observe that two solitons merge into a single short-lived pulse (i.e., strong merger regime)^25–27^ and annihilate each other. In particular, for the shown example, four main solitons dominate transition to the fast-slip phase and they prevailingly move with the velocity of 0.03C featuring slow propagating velocity. Here, C is the maximum allowed velocity and we use a normalized value of C≡1. For mica this is on the order of a few km/s corresponding to surface waves (see Methods section for measuring C - Fig. S9). Tracking of soliton fronts unravels the attractive interactions of colliding solitons (Figs 5 and S7). This attractive interaction leads to a “sudden” increase in the front velocity up to ~0.9 C - in less than a few hundred nanoseconds implying an abrupt local acceleration up to ~10^9^ m/s^2^, four orders of magnitude higher than reported in regular laboratory stick-slip experiments that employ low frequency load cells and gauges^28,29^. Particularly, accelerated soliton fronts result in faster annihilation and eventually yield a shorter fast-slip phase (Fig. 5), hence quickening the weakening phase of micro-failure events associated with displacement bursts. The observed Λ-shape collision of solitons and their annihilation are universal features of the studied events in our experiments and modulate the relaxation of the system across the transition between phase (I) and fast-slip (II) phases. The attractive interaction of soliton fronts may occur due to the nonlocal nonlinear nature of the medium in which the response at a certain site is transferred to the surrounding regions and induces a spatially long-range response of the medium^30,31^. The lifetime of fast fronts (maximum ~800 ns) is shorter than the lifetime of slower fronts (maximum a few micro-seconds). Due to merging of the solitons, the evolution path of the system after crossing the peak of <k> is reversed and the system approaches another (ground) state in which all sites point inward (Figs 4b and S8).

**Figure 6:**
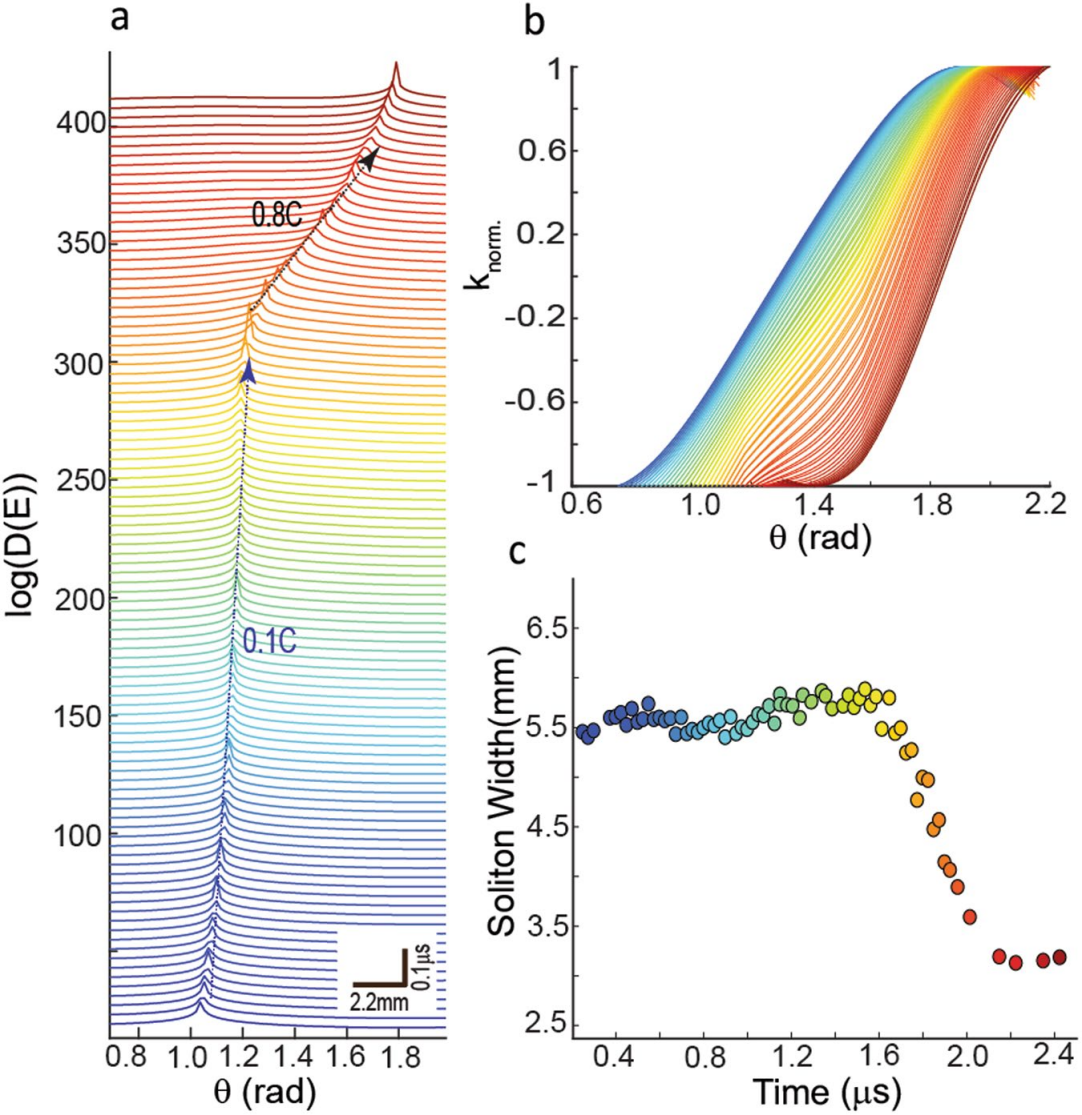
Squeezing a moving kink’s width as it accelerates. (**a**) Velocity transition of a moving kink with an average velocity of 0.1 C to ~0.8 C in transition from phase I → II. The color denotes the passage of time. **(b)** Normalized soliton profiles with similar color coding as (**a**). The Lorentz contraction is evident in the profiles as a decrease in the width of the profiles (dashed arrows) when the speed of propagation *v* is increased. We have shifted the profiles horizontally to show the effect of squeezing on the width of soliton. **(c)** The width of the soliton shrinks up to ~45% when the relative propagation velocity increases ~6 times of its initial velocity (see Fig. S8 as another example).

A striking feature of moving solitons as proposed using several analytical solutions^20–22^, is that solitons with velocities approaching the maximum allowed velocity in a given structure exhibit relativistic features. In Fig. 6 – also see Fig. S8 - we show the trajectory of a soliton in transition from phase I to II where a sudden jump in the front velocity of up to 6 times the initial velocity is observed. After this sudden acceleration, the velocity of the soliton approaches ~0.8 C and its width *w* shrinks by ~45% of the initial length. This squeezing of soliton characteristic length is consistent with Lorentz contraction and hints at the existence of relativistic features occurring during the displacement bursts.

## Discussion

Our results provide new fundamental insights into microscopic dynamic failure under a sharp indenter tip. We presented experimental evidence of short-term strain disturbances in the form of moving solitons in the course of displacement-bursts. Tracking the motion of single solitons unraveled their abrupt acceleration that occurs during the fast weakening phase - the most critical phase of any unstable material failure. Motivated by our results, we argue that existence of solitons as the shear components to an evolving k-lattice are analogous to the observed rupture fronts moving along an active frictional interface. From this point of view, the propagated soliton along a non-deformable chain represents a single moving shear front as it has been represented in recent experiments^8,32^. Therefore, we speculate that multiple nucleated fronts along an interface, in contrast with a single hypocenter, can result in an abrupt local accelerated scenario with magnitudes much higher than global observed values of the slip acceleration. Existence of a short-term relativistic state in motion of solitons implies increasing the energy of moving defects which in turn modifies the energy budget of the system. For a soliton moving with 0.8 C, the consumed energy is 67% higher than for slow fronts (a few percent of C) and effectively acts as a non-trivial energy sink of the system.

In summary, our work indicates that the introduced framework to analyze recorded ultrasound emissions provides a versatile platform to study displacement bursts during contact-based failures and establish novel methods for probing dynamic defects.

## Materials and Methods

### Experimental procedures

We indented Muscovite Mica specimens (from Princeton Scientific Corp). The indentation of mica sheets was performed parallel to [001] direction. We first suspended a thin sheet of mica on a ring–like structure of ultrasound sensors and carefully glued on the sensors. Then, we mechanically exfoliated the mica by peeling sheets from the top of the specimen. We repeated this process 10 to 15 times to achieve a thin layer (~5–20 µm) of the mineral sheet suspended on the sensors. The central indentation was performed using a micro-indentation instrument (DUH-211). We used different scenarios of loading with loading rates of 3mN/s. Our focus here was on the recoded acoustic emissions during loading or creep stage of the tests. In order to detect ultrasound emissions, we utilized piezoelectric sensors with frequency bandwidth of the transducers of 0.1 to 2.5 MHz. In the primary set-up (Figs 1 and 2), we used 8 sensors; to confirm the results we employed a second set-up with 16 sensors. The acoustic (ultrasound) signals-i.e., AE events- are first pre-amplified at 60 dB, before being received and digitized. In Fig. S1, we have shown some of the recoded waveforms. The source of emissions are two folds: dislocations and micro-cracks. To differentiate the sources and assuming that each event is corresponding to a single point-like event, we can use a source mechanism algorithm to distinguish the nature of events (similar to ref.^12^). While this method is an approximated technique, however might be supported by inferring activation energy of an event using duration of events in the rising (phase I) and weakening phase (phase II). Regardless of classification of events, we use the term of “crackling” to both of the categories and note that the obtained <k> -profiles do satisfy two main components of the deformations: non- deviatoric deformations are manifested as pure expansion or contraction and mixed deformations where both isotropic and deviatoric components are present. Deviatoric components are due to excitation of (shear) solitons and when *E* → 0 the main deformation mode of the chain is governed mainly by moving kinks. To have a k-chain without solitonic state –i.e., pure isotropic event- one should transit to the second phase without inducing any non-linearity to <k(t)> in vicinity of the k_max_. This is equal to pushing the effective temperature in thermal activation model of events to zero (T → 0)-see section 3 of Supplemental Information.

### Analysis of emitted ultrasound waves

We map the recorded acoustic emission array (i.e., array time series for each event) to a mathematical graph. We use a previously developed algorithm to construct the mathematical graphs from our reordered acoustic emissions with a fixed number of nodes^9,13,33,34^. The main steps of the algorithm are as follows (Fig. 1) (1) the waveforms recorded at each acoustic sensor are normalized to the maximum value of the amplitude at that site. (2) Each time series is divided according to maximum segmentation, such that each segment includes only one data point. If we want to use correlation measure, we need to increase the segmentation window. The amplitude of the *j*th segment from the *i*th time series ($1\le i\le N$) is denoted by ${u}^{i,j}(t)$ (in units of mV). *N* is the number of nodes or acoustic sensors. Here, we set the length of each segment as a unit with a resolution of 25 ns (3) ${u}^{i,j}(t)$ is compared with ${u}^{k,j}(t)$ to create links between the nodes. This means If $d({u}^{i,j}(t),{u}^{k,j}(t))\le \zeta$ (where *ζ* is the threshold level) we set ${a}_{ik}(j)=1$ otherwise ${a}_{ik}(j)=0$ where ${a}_{ik}(j)$ is the component of the connectivity matrix and $d(\,\cdot \,)=|{u}^{i,j}(t)-{u}^{k,j}(t)|$ is the employed *similarity metric*. With this metric, we simply compare the normalized amplitude of sensors in the given time-step. The employed norm in our algorithm is the absolute norm. (4) Threshold level (*ζ*): To select a threshold level, we use a method introduced in^33,34^ and references therein) that uses an adaptive threshold criterion to select *ζ*. The result of this algorithm is an adjacency matrix with components given by $a({x}_{i}(t),{x}_{k}(t))={\rm{\Theta }}(\zeta -|{u}^{i,j}(t)-{u}^{k,j}(t)|)$. Here ${\rm{\Theta }}(\,.\,)$ is the Heaviside function. The constructed lattice is characterized by the average of all nodes’ degree (<k>), where the degree, *k*_i_, is the number of links formed between each node *i* and all other nodes in the system. For each node we assign ${s}_{i}=sign(\frac{\partial {k}_{i}}{\partial t})$ and then *s*_*i*_ = ±1. With this mapping, each node in a given time step acquires one of the states (outward: → or inward: ←).

The employed method can be understood from the perspective of Fourier transform of equal-time correlation measure over the waveforms in a given time-window. Note that the measure of correlation functions is the root of many scattering experiments and Fourier transform of equal-time correlation function of a certain quantity –such as electron density or amplitude of observer -is the intensity of the scattered injected energy^35^. The employed algorithm quantifies the intensity of equal-time similarity between nodes; higher value of <k> indicates higher closeness in a given time; the method is more robust for a limited number of observers than the Fourier transform which is more suitable for a large number of points^18^. In short, in a given time interval, k-parameter represents the abundance of a certain energy level as encoded in waveforms which have been recorded by a spatial distribution of observers (nodes). Furthermore, comparison of the average of power spectrum as the result of time-domain spectroscopy-$P\equiv {|FT|}^{2}$-over a broad frequency range $< P{ > }_{\omega }$- witch shows that rising section of the $< P{ > }_{\omega }$ coincides with the second phase in evolution of <k(t)>_*x*_.

In all the reported cases in this study, we used a fixed number of sites (N = 300 nodes) with interpolating the connectivity matrix, resulting the lattice constant as *a* = *0.2386* *mm*. To confirm the results, we also used a second set-up with 16 transducers where we indented a similar Muscovite Mica on a substrate of aluminum plate where the lattice parameter for N = 300 nodes was *a* = *1.03* *mm*. To measure the velocities of a moving kink –which are shear solitons for the k-lattice- we track soliton movement in $\theta -D(E)$ space where we shift D(E) in a fixed time-step- see Figs 4–6. The measured velocities are in units of $\frac{{\rm{\Delta }}\theta }{{\rm{\Delta }}t}$ and we convert Δ*θ* (in radian) to Δ*l* (travel length in millimeter) based on the employed lattice spacing. The estimated velocity from this method indicate that the (apparent) propagation velocity in $\theta -D(E)$ space can be higher than p-wave velocity of the mica (see Fig. S9). This is due to the projection of velocity vectors to $\theta -D(E)$ where the mass-center of a soliton usually is in oblique angle with the circumference of the k-chain –as the θ direction in our system. Therefore should not be interpreted as hypersonic regime of propagation. We report relative velocity of a moving kink relative to maximum allowed velocity (C). To estimate the maximum allowed propagation velocity of a kink, we use the Lorentz contraction relation $\frac{\xi }{{\xi }_{0}}=\sqrt{1-{(\frac{v}{C})}^{2}}$ where we could precisely measure the characteristic length of the soliton (ξ) before and after dramatic change of the velocity.

### Calibration of k-chains with mechanical impulsive sources

To associate <k(t)> with a physical source parameter, we used recorded un-amplified AE signals from known sources of impulsive compressive and shear loads recorded on a ring-like array of ultrasound transducers (Figs S3 and 4). To generate compressive stress, we used a spilt Hopkinson pressure bar apparatus where an impulsive stress pulse is generated by a cylindrical steel projectile (the striker bar). The flying striker bar impacts an incident bar of identical material and diameter (Fig. S4a) and induces a strain rate on the order of ~10^1^s^−1^. The source signal is transferred through the incident bar and impacts a second bar which we mounted with 6-array Piezo-electric transducers. Using Dynamic linear strain gauges and knowing that the apparatus generates compressive stresses, we can compare <k(t)> with the strain gauge records (i.e., strain and stress on the bar). Here the source has the shape of a Gaussian function with superimposed oscillations due to the resonance of the bar (Fig. S4c). The calculated <k(t)> -phase I of the evolution-is compared with recorded strain (Fig. S4d), indicating that <k> -profiles capture main features of the stress change. To compare the <k(t)> with shear sources, we used similar unamplified ultrasound transducers as well as dynamic strain gauges and an accelerometer while two halves of the saw-cut Westerly granite slide on each other (Fig. S3). We confirmed that the calculated <k(t)> represent dynamic stress change on the interface due to abrupt stress drop (stick-slip tests). Neither measured acceleration nor measured velocity represent the <k(t)>. The form of the <k(t)> profiles measured resemble slip within granite blocks during propagation of rapid macro-rupture fronts.

## Supplementary information

Supplementary Movie 1

Supplementary Movie 2

Supplementary Information

## Data Availability

The data presented in the figures and that support the other findings of this study are available from the corresponding author on reasonable request. The k-chain implementation using recorded ultrasound emissions is available upon reasonable request.
