# Supplementary material for: Solitonic State in Microscopic Dynamic Failures
Source: Sci Rep. 2019 Feb 13;9:1967. doi: 10.1038/s41598-018-38037-w (PMC6374453; doi:10.1038/s41598-018-38037-w)
Supplement: Supplementary file 3 — Supplementary Information [file 41598_2018_38037_MOESM3_ESM.docx]

*Supplementary Materials:*

Solitonic State in Microscopic Dynamic Failures

*H.O. Ghaffari ^1^* * *,W.A.Griffith^2^* and M*.Pec^1^*

*^1^ Department of Earth, Atmospheric and Planetary Sciences, Massachusetts Institute of Technology, Cambridge, Massachusetts, USA*

*^2^ School of Earth Sciences, Ohio State University, Columbus, Ohio, USA.*

**Correspondence to:* [*hoghaff@mit.edu*](mailto:hoghaff@mit.edu)

1. **Experiments**

We have done several experiments using commercial ultrasound transducers (pico-sensors, and physical acoustic system-PAC-sensors). In the following, we report three different set-ups of mechanical excitation of sources. While in all indentation tests we employed amplified PZTs , in experiments pertaining impulsive mechanical loadings (compressive or shear) ,we used non-amplified PZTs. This allows us to study the recorded signals without concern on losing the data due to “clipping” of waves which usually occurs for amplified waves emitted from high energy sources. Also the effect of amplifier’s response on recorded signals is eliminated.

The summary of experiments are as follows: (1) Indentation of suspended thin Mica films over 8 PZTs (2) Split-Hopkinson bar in studying compressive waves (without sample) and using dynamic strain gauges and recording axial strain field (Fig.S.4) (3) dynamic shear tests on PMMA blocks under uniaxial test and employing an accelerometer to record acceleration of the moving interface with recording un-amplified waveforms (Fig.S.3).


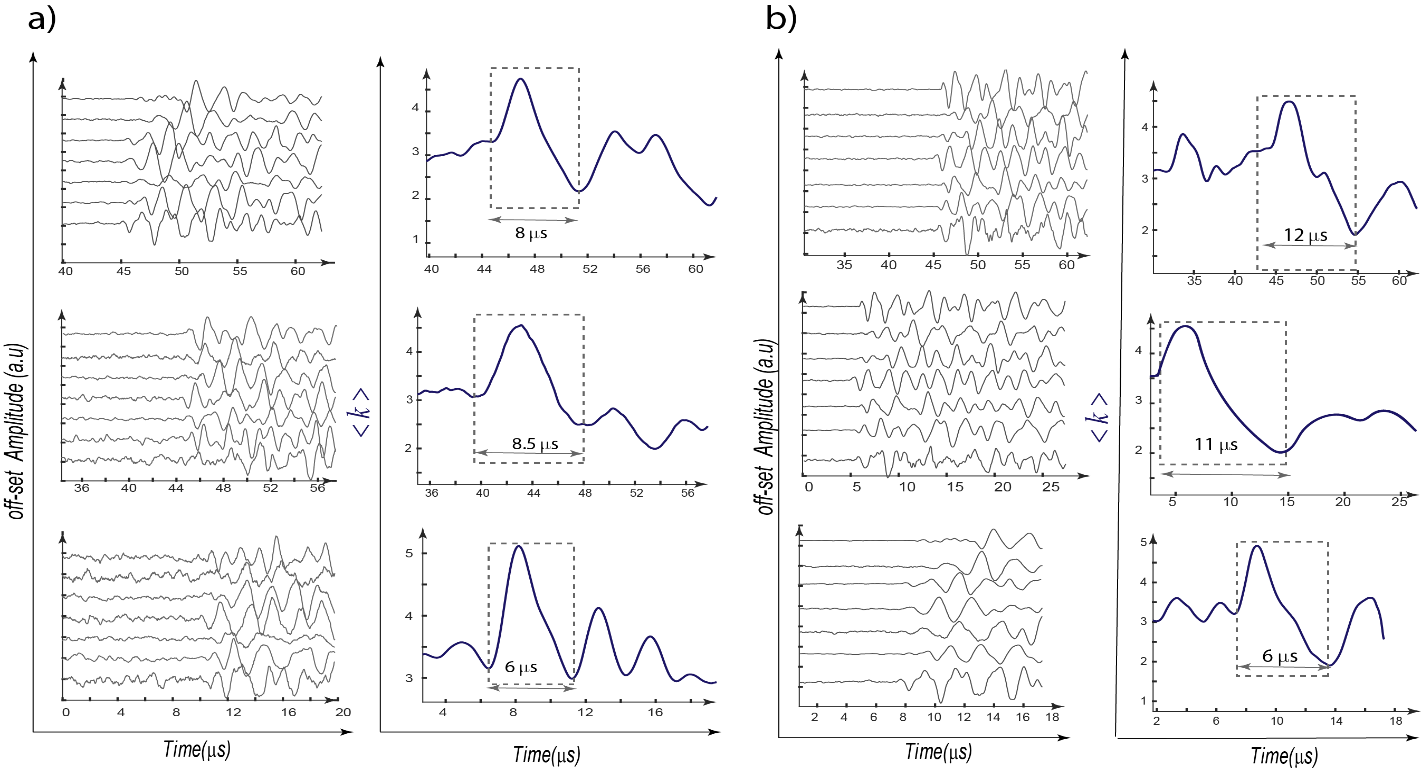


**Fig.S1.** Some of recorded waveforms due to distinct AEs in one of our experiments. We show the scaled waveforms and corresponding <k(t)> profiles . In Fig.2e of the main text, we have shown some of the <k>-parameters corresponding to these waveforms.


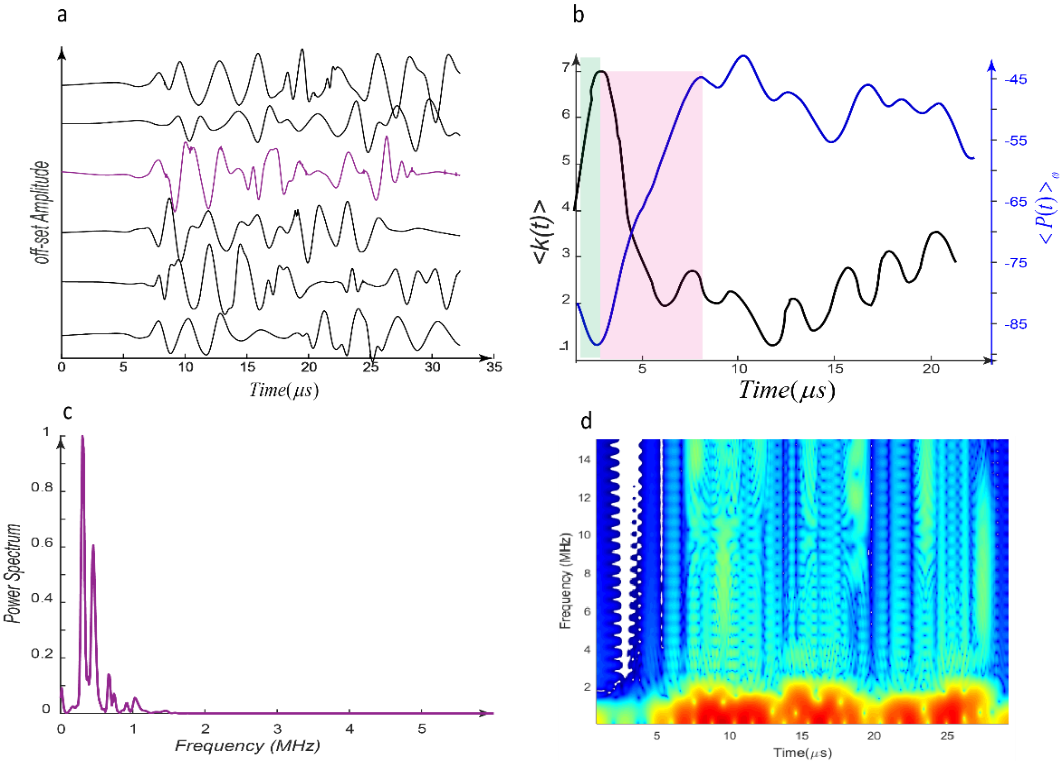


**Fig.S2.** **(a)** **A recorded event from indentation of mica- we show 6 waveforms**. **(b)** The <k(t)> parameter is calculated and compared with the average of $P\equiv\left|FT\right|^{2}$over a broad frequency range of 1kHz to14 MHz . The average power spectrum is calculated for the shown purple signal $<P>_{\omega}$ . The rising section of the $<P>_{\omega}$ corresponds to extended high frequency section of the spectrum-see the spectrogram of the waveform in panel **d**- and this coincides with the second phase in evolution of <k(t)>.

**Series of frictional experiments (shear sources):** Previously, we stablished that <k(t)> could share significant similarity to evolution of the physical recorded strain fields in stick-slip experiments in rock samples (FigS3 a-b)-Also see References [1] and [2]. An additional new experiment was carried out by employing two PMMA blocks on top of each others with an angle of ~20 degree where we used a uniaxial loading configuration on saw-cut cylinders of PMMA. We employed an accelerometer and 7 PZTs where we did not amplify the signals (Fig.S3c-f). With using simplex algorithm in determining the source location of the excited event (usually we get a single event in this case) and assuming a velocity model, we could define the source location of the triggered event. After employing k-transform procedure, the recorded waveforms were transferred in a single <k(t)> profile. Comparing the acceleration and the calculated displacement from the recorded acceleration indicated that <k(t>) does not resemble either of these parameters but is quite similar to our previous measurement on shear strain evolution on rock-rock frictional interfaces (Fig.S3a) . The Duration of the fast-weakening phase in the latter case is up to 60-70 µs while this interval is much shorter for rock-rock typical signals ~15-25 µs (Fig.S3b). Please note that the duration of fast-slip phase in most of the recoded events in our indentation tests is about ~3-12µs (Fig.S.1) much shorter than the rock-rock or PMMA-PMMA frictional tests.


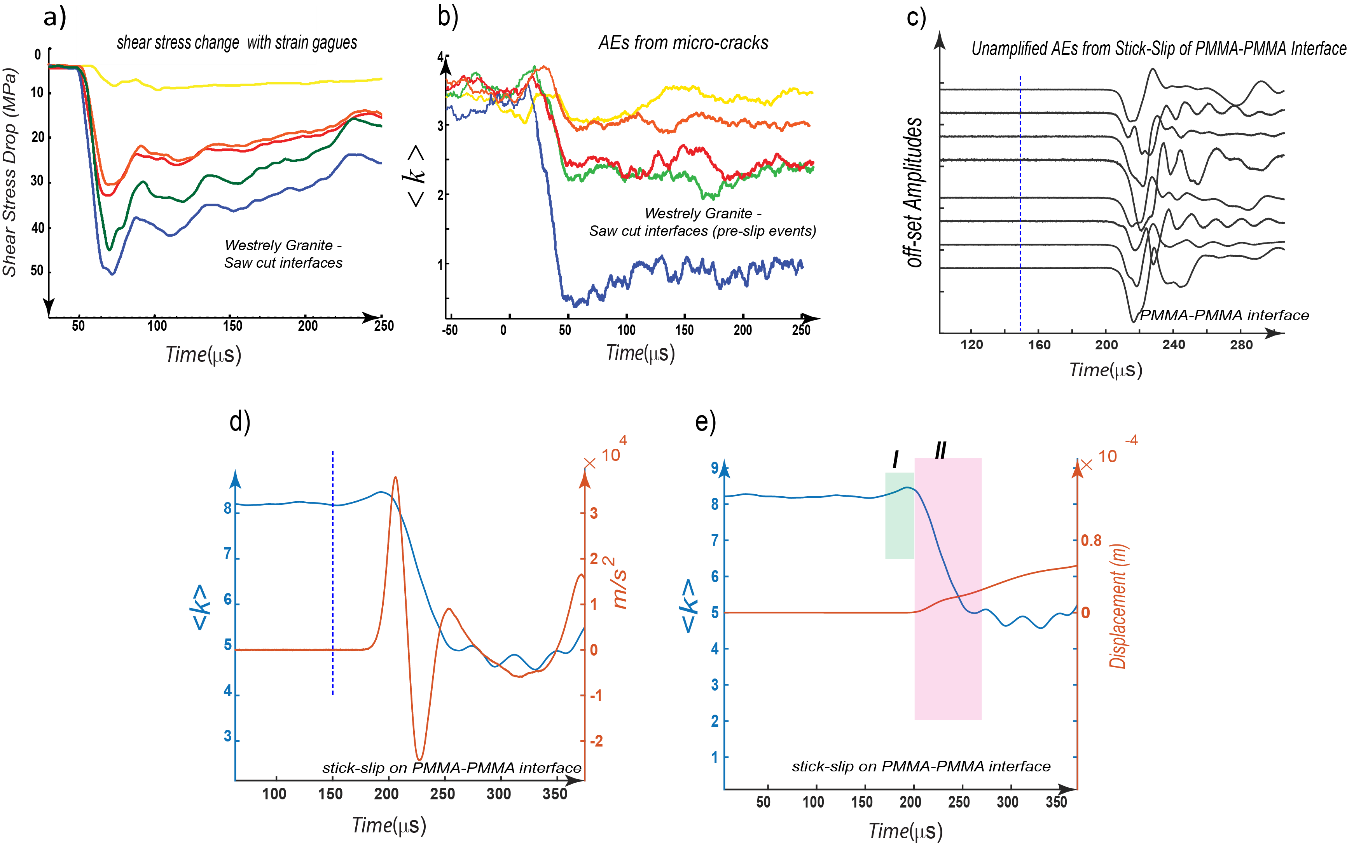


**Fig.S3.**  **Shear source and k-chains (a)** the **measured dynamic shear stress** evolutions in two different shear-experiments : in the first case we recorded the (dynamic) strains while two halves of the saw-cut Westerly granite slide on each other leading to macro-slips (major stick-slip ). We show 5 stick-slip events with the calculated shear stresses based on the measured strains. Wheatstone bridge strain gages are employed to record the dynamic (shear) stress change at 10MHz (the slope of the fault was 60 degree with horizon (see details in [1]) **(b)** The calculated <k(t)> based on micro-cracks prior to major stick-slip experiments based on recorded ultrasound excitations (AEs) [2] . The main phases of <k(t)> share similar evolutionary trends with (a) . **(c) The second shear-test: The recoded unamplified waveforms -**after stacking-under uniaxial stress while two halves of the PMMA-PMMA slide on each other. The blue dotted-line is the arrival of p-wave. **(d-e)** The calculated <k(t)> based on unamplified signals and super-imposed acceleration and displacements. The calculated <k(t)> represents dynamic strain change due to stick-slip and neither of measured acceleration or velocity represent the <k(t)>.

We can map the uni- or bidirectional rupture fronts –propagating along the frictional interface- on to a ring. Here we virtually glue site 1 to 8 (periodic boundary condition) and then a rupture front is mapped as a rotational front along the circumference of interface-ring. We can –also-map back the orbiting fronts along the ring on an interface. Therefore, the propagated fronts along the chain on average represent evolution of an interface. Please note that here the ring is a solid interface and does not deform; i.e., it does not involve any non-deviatoric components. In the case where the ring does include isotropic terms (compressional or tensile), the deformation of the chain involves three components which could occur simultaneously.

**Compressive-impulsive sources:** In a series of experiments, first we used recorded un-amplified AE signals from known sources of impulsive compressive recorded on an array of ultrasound transducers; (Fig.S.4). Using the spilt Hopkinson pressure bar apparatus, an impulsive stress pulse is generated by a cylindrical steel projectile (the striker bar). The flying striker bar impacts an incident bar of identical material and diameter (Fig.S4.a). The source signal is transferred through the incident bar and impacts a second bar which we mounted with 6-array Piezo-electric transducers. Using dynamic linear strain gauges and knowing that the apparatus generates compressive stresses, we compare the sensors response with the strain gauge records (i.e., strain and stress on the bar). Here the source has the shape of a Gaussian function with superimposed oscillations due to the resonance of the bar (Fig.S4b). The experiment is similar to employing amplified sensors in higher energy ball-drop tests in order to calibrate PZTs where a Hertzian contact force is assumed as the source and the response of the instrument is filter out (convolving) with assuming the response of the media. As we have shown in Fig.S4b the maximum amplitude of the first motion of the PZT coincides with the maximum linear strain –prior to distortion of the signal by the resonance waves of the vibrating bar. The compressive loading portion in the impulsive source is recoded with an initial rising phase in the PZT’s signal confirming that the employed PZTs are positively polarized to compressive stresses. Then, the calculated <k(t)> -phase I of the evolution-is compared with recorded strain (Fig.S4d), indicating that <k>-profiles capture main features of the stress change.

*
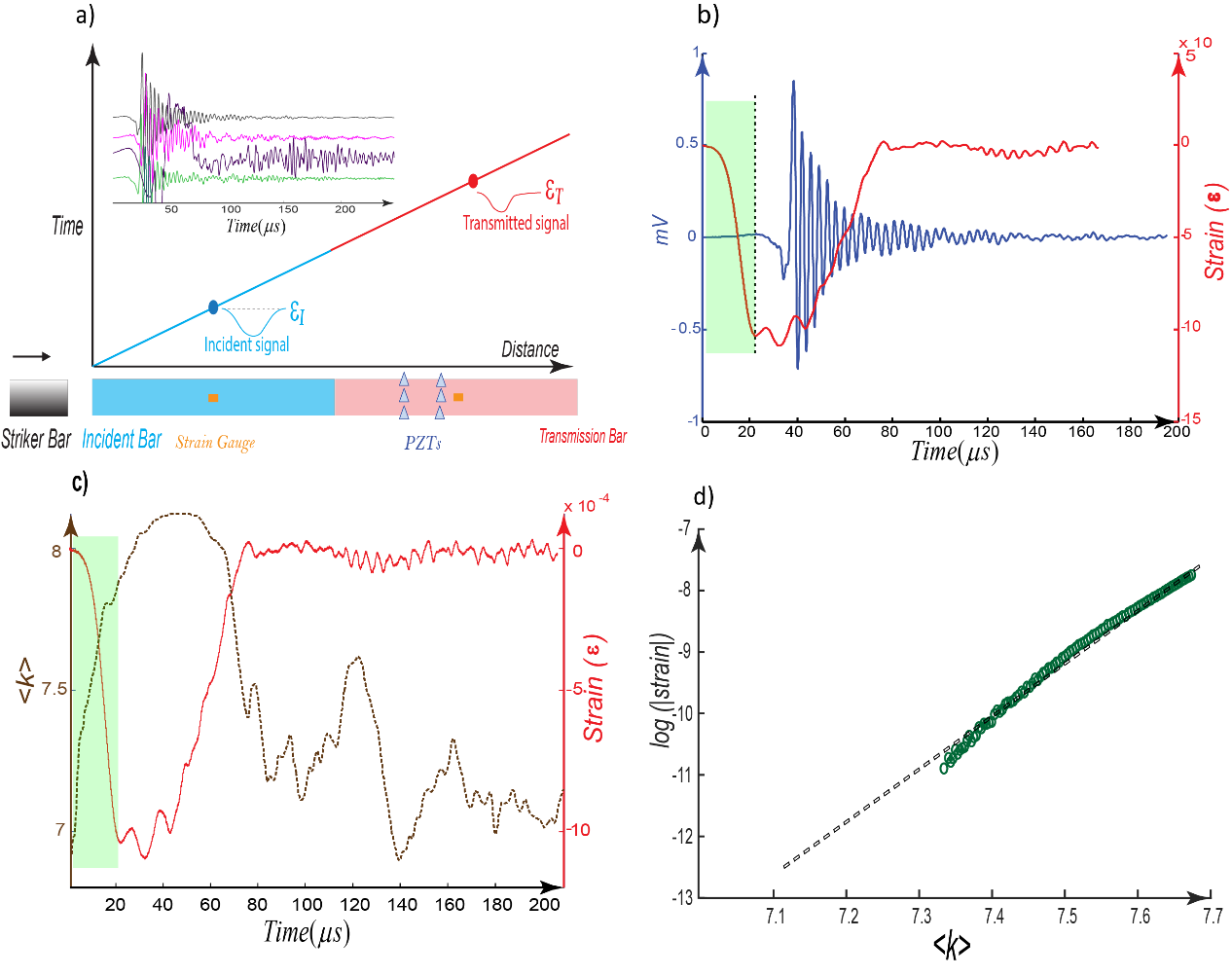
*

**Fig. S4.** **Compressive Source** **and k-chains.**  Calibration of the employed ultrasound transducers with known source and (dynamic) strain gauges using impulsive dynamic loading tests (generated by split Hopkinson Pressure bar). (a) Here we show the response of the sensors to a controlled source tuned by the velocity of striker bar and pulse shaper. We used two dynamic strain gauges in incident and transmitted bar. We also used 6 unamplified piezo-electric transducers (PZTs) with the frequency bandwidth of the transducers from 0.2 to 1.3 MHz. The transducers were mounted on transmission bar in different positions. (b) Transmitted compressional signal (in red and negative sign of strain) superimposed on the recorded PZT signal. (c) Double plot of the recorded strain gauge and calculated <k> parameter. (d) Semi-logarithmic scale of<k> vs. strain during the rising phase of strain-time prior to effects of bar-resonance.

1. **On Energy of k-Chains**

In the main text, we evaluated the quasi momentum- energy space where we used kinetic energy term and ignored other energy terms. Here we point out some other possibilities in estimation of interaction (potential) field and using spectral analysis to analysis the introduced (active) 1-d lattices. In Fig.S.5, we show that the transition from phase I (as the initial strengthening phase) to the second phase (weakening phase) is accompanied with transition to higher energy (higher frequency component) of the waveforms. In particular, in Fig.S5b, we see clear energy bands in *<k>- energy* parameter space for three different events. Here we have calculated the signal power (from Fourier transformation analysis) averaged over the frequency range of 0.1MHz -2MHz . The transition zone with energy gap of 2Δ is the manifestation of the solitonic state. One can assume that <k> is the local-control parameter of the system (=k-chain) and is ramped from <k>_0_ (initial value) to <k>_I_ (see Ref.13 of the main text).


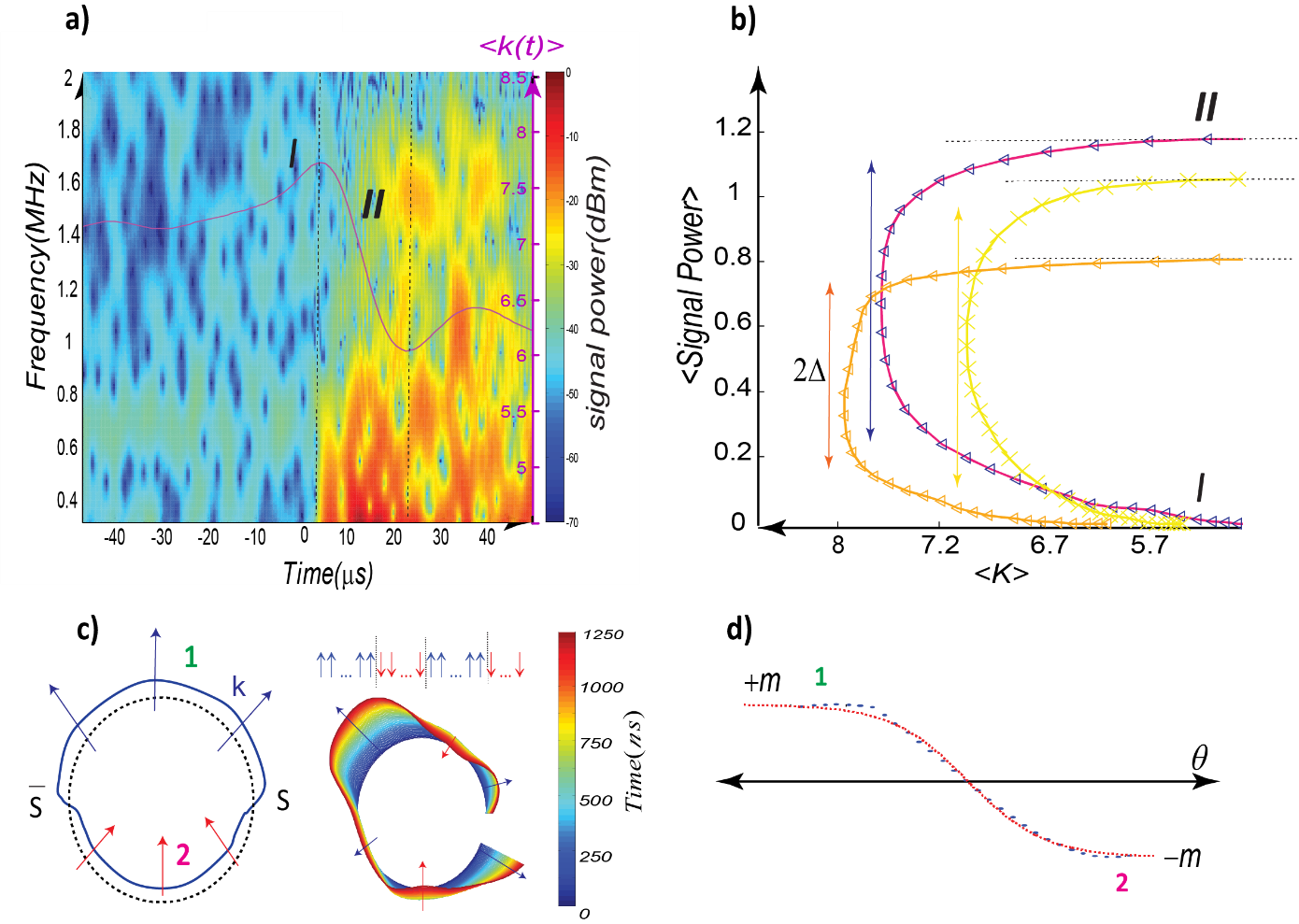


**Fig. S5.** **a)** **Study of energy through spectral analysis versus <k> in a given waveform.** Comparison of the power spectrum as the result of time-domain spectroscopy-$P\equiv\left|FT\right|^{2}$- with shows that high frequency section coincides with the second phase in evolution of <k(t)>*_x_*. (b) *<k>-* $<P>_{\omega}$ parameter space for three different events-$<P>_{\omega}$ : the average of power spectrum as the result of time-domain spectroscopy-$P\equiv\left|FT\right|^{2}$- The transition zone with energy gap of 2Δ is the manifestation of the solitonic state.


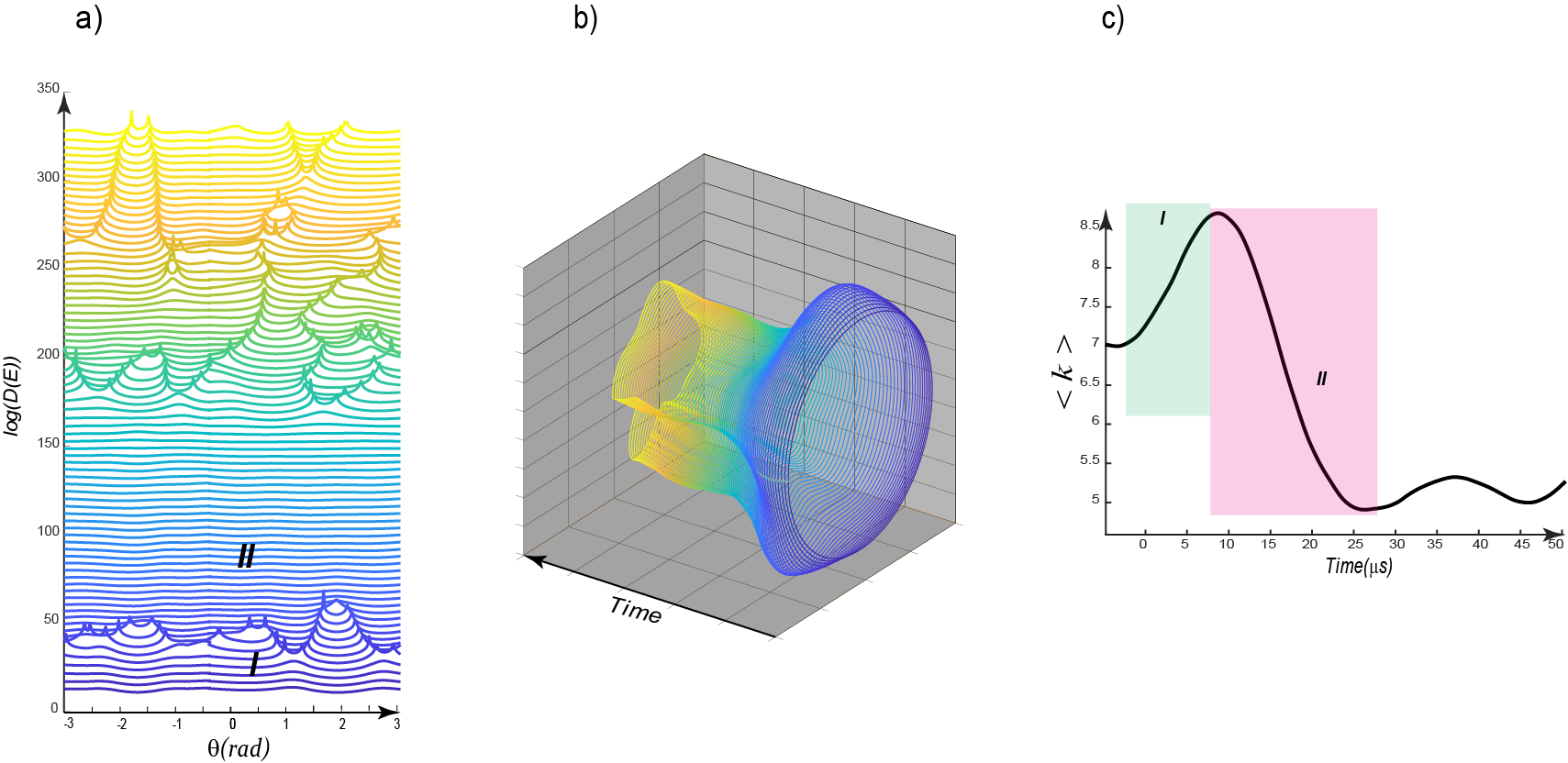


**Fig.S6.** **3D visualization of a k-chain’s evolution (**see the supplementary movies 1 and 2).


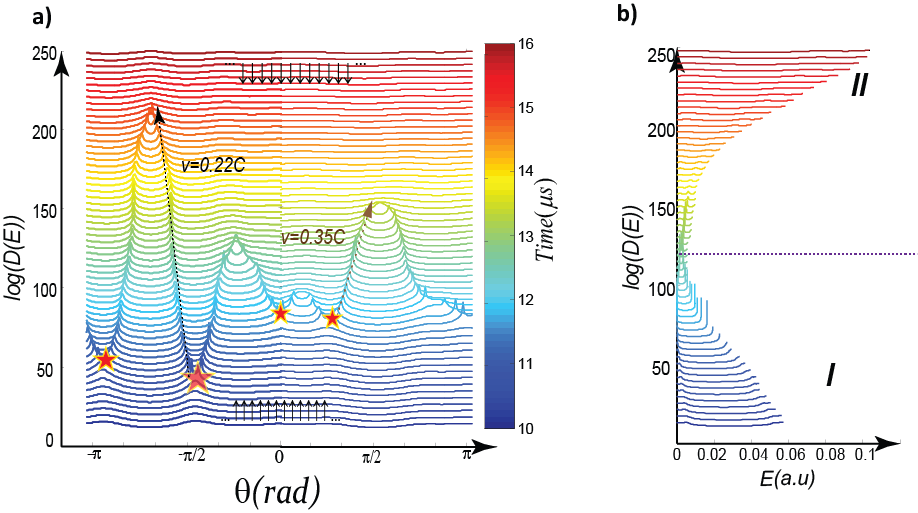


**Fig.S7.** **Formation of 4 moving solitons** along the chain in transition of phase I to phase II. One clearly observes proliferation, colliding and merging of fronts. **E** is the kinetic energy of shrinking (folding) or expanding sites. Approaching E→0 in (b) indicates that the solitons (=moving kinks) are dominant state of the deformation of the chain.


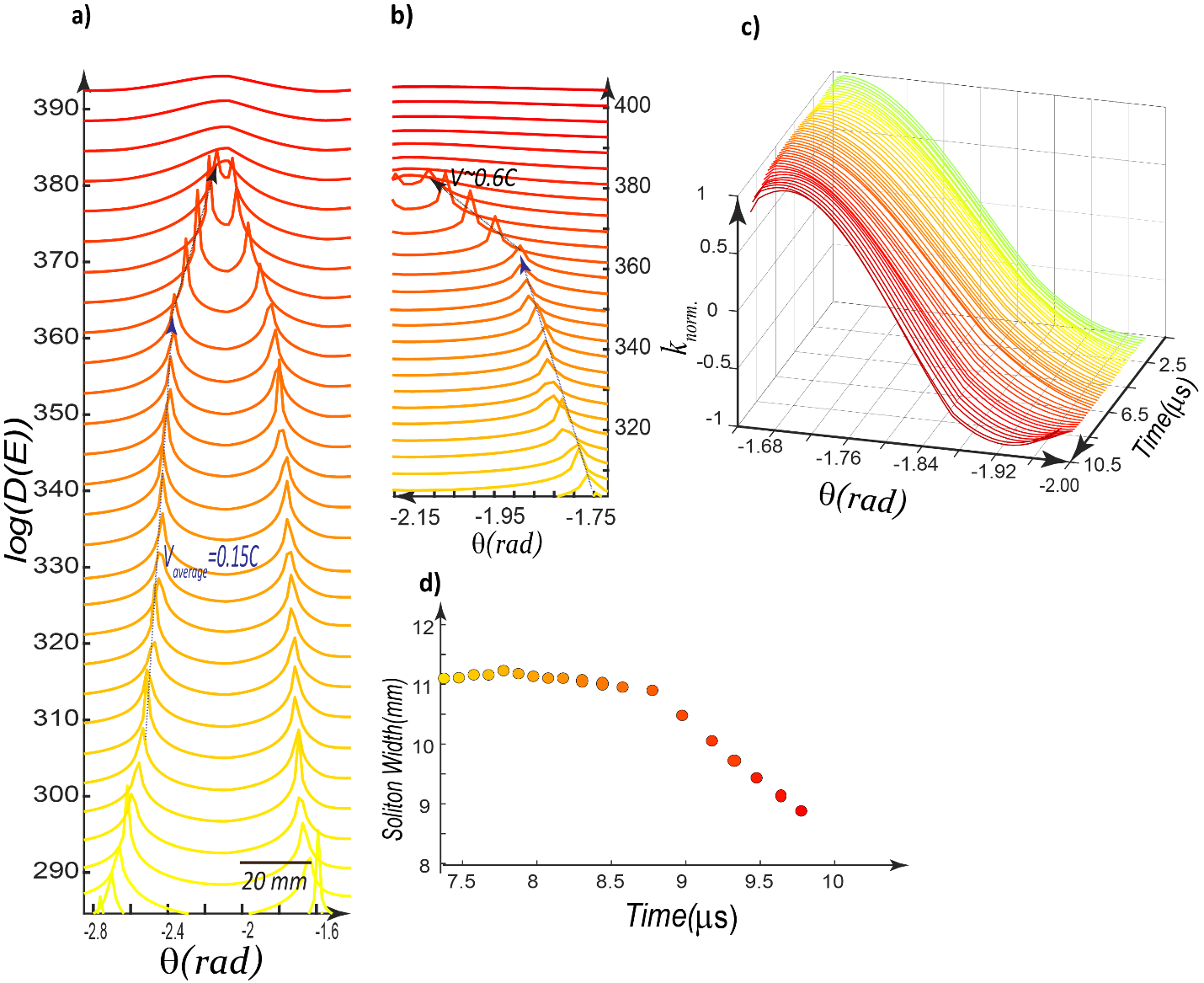


**Fig. S8| Observation of Lorentz contraction in an accelerated soliton. a,b)** Velocity transition of a moving kink with velocity of ~0.15C to ~0.6C in transition from phase I→II, leading to a fast acceleration. The color denotes the passage of time. The density profiles are in 0.1 µs increments and are shifted vertically for clarity. **c)** Normalized soliton profiles with similar color coding as (a&b). The Lorentz contraction is evident in the profiles as a decrease in the width of the profiles when the speed of propagation is increased. **d)** The width of the soliton shrinks when the relative propagation velocity increases ~4 times its initial velocity.


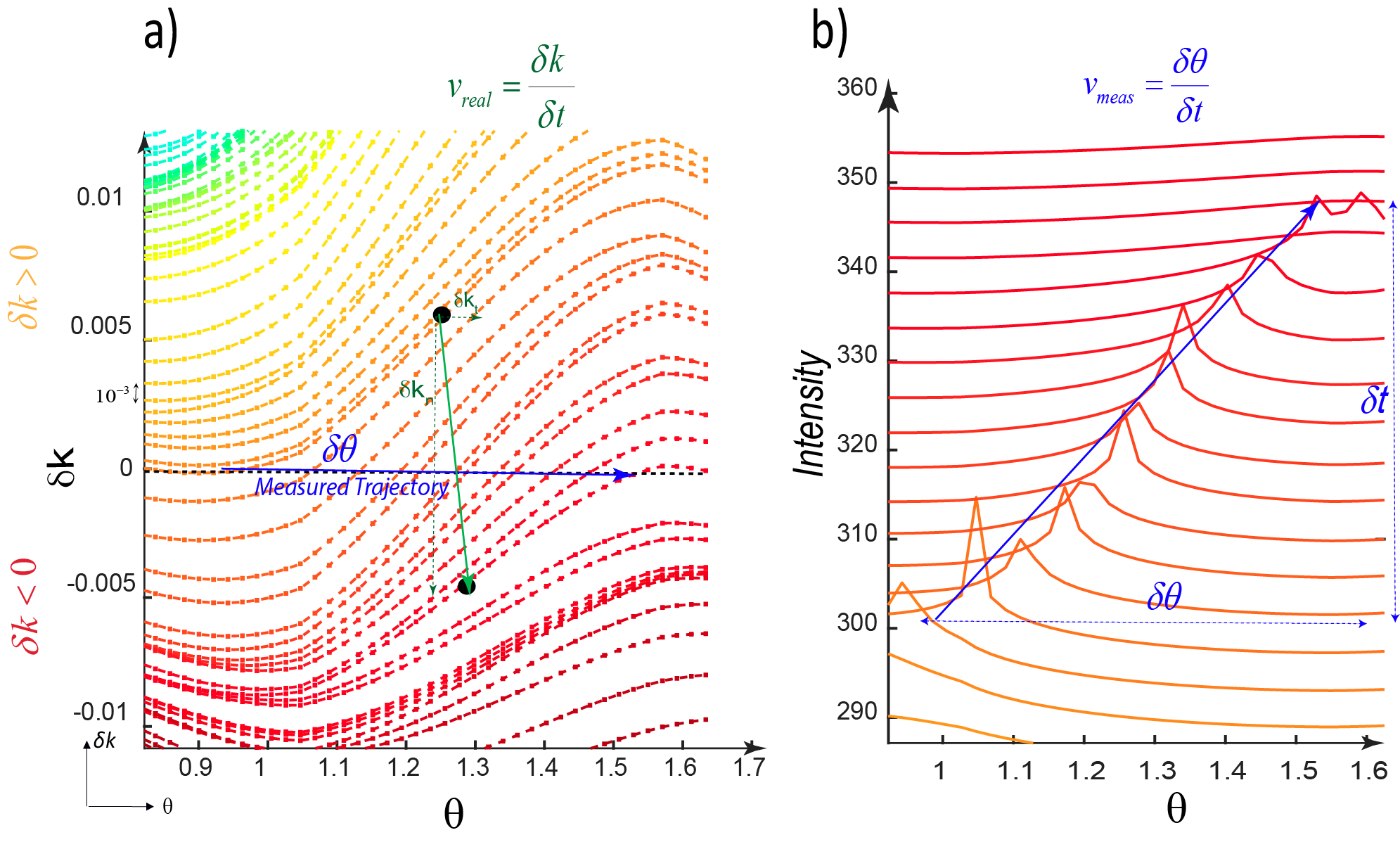


**Fig. S9| Solitons:** We define a soliton when a profile of $\delta k(\theta)$ crosses$\delta k=0$. The rate of moving *“zero”* modes,$\delta k=0$, has been reported as soliton velocity ($v_{meas}\equiv\frac{\delta\theta}{\delta t}$). The true velocity of the soliton is given by $v_{real}\equiv\frac{\delta k}{\delta t}$which usually is smaller.

1. **K-lattices : Relationship with *Fermi-Dirac* distribution and interatomic distance-force curves**

Here we provide a semi-theoretical proof of k-chains as a measure of (mean) strain field. To do this, we map the k-chains onto a free electron gas model and use *Buehler-Gao’s* [3] interpretation of interatomic force (hereafter B-G’s model). To start we assume a configuration of a k-chain prior to onset of a kink excitation. The clusters –or communities [Ref. 14, 20 of the main text]- of the similar sub-energies carry out the energy of the chain; hence we define a certain number of the main energy levels (*m*) and as a first order approximation, we consider non-interactive energy levels. Each energy level does include sub-level energies which through the links form a community; in non-interactive energy clusters the links are purely within main energy levels and do not extend to other energy levels (“*ideal gas*” analogy). We assign a kinetic energy to each level$\varepsilon_{i}$ and abundance of the *i*^th^ state, i.e., *k_i_* , which is proportional with the occupation number of the *i*^th^ level$n_{i}=\frac{k_{i}}{\sum_{m} k_{i}}$ and summation goes over *m-*main energy levels. Next, we use a probabilistic argument that probability of finding an energy level with a higher value declines exponentially proportional with the energy level. Furthermore, we can define a maximum kinetic energy µ as a reference point; this is the allowed maximum kinetic energy level (in analogy with the Fermi level). The occupation number of *i*^th^ energy level is, therefore, given by the Fermi-Dirac distribution [4]: $n_{i}=\frac{1}{e^{\frac{(\varepsilon_{i}-\mu)}{k_{B}T}}+1}$ in which $n_{i}$is the occupation number of the *i*^th^ energy level, $\varepsilon_{i}$is the kinetic energy, µ is the internal chemical potential (at zero temperature, this is the maximum kinetic energy, i.e. Fermi energy), T is the absolute temperature k_B_ is the Boltzmann constant. Now, we relate the above argument to stress-strain curve (i.e., force-distance). To this end, we use *Buehler-Gao’s* interpretation of interatomic force versus atomic separation *r* which is given by (3): $F(r)=k(r-r_{0})\left[\frac{1}{e^{\Xi(r-r_{c})}+1}\right]$where the parameter *r*_0_ refers to the nearest-neighbor spacing of atoms. Assuming that the spring constant *k* is fixed, the *F(r)* has two other free parameters, *r*_c_ and Ξ. The parameter *r*_c_ corresponds to the Fermi energy in the Fermi–Dirac function µ and denotes the critical separation for breaking of the atomic bonds. The parameter Ξ corresponds to the temperature in the Fermi–Dirac function and describes the intensity of smoothing at the breaking point. The exact mapping can be achieved by [3] $r\leftrightarrow\varepsilon_{i},\Xi\leftrightarrow\frac{1}{k_{B}T},r_{c}\leftrightarrow\mu$. Thus, the evolution of *i* ^th^ energy level in a k-chain is related to interatomic separation as follows: $r_{i}=r_{c}+k_{B}T\log(\frac{\sum_{m} k_{i}}{k_{i}}-1)$which indicates that the growth of the energy levels are exponentially proportional with the interatomic separation and therefore cohesive stress (Fig.S.10). Interestingly, the later conclusion confirms our calibration results upon to the failure point in which a semi-logarithmic change of the strain is scaled with the variation of node’s degree (Fig.S.4d). It is noteworthy that increasing effective temperature in B-G’s model is in direct connection with the smoothness of the stress-strain curve in vicinity of the failure point; increasing effective temperature yields smoother curvature in failure point. Knowing that the solitionc mode -including number of solitons and their interactions -significantly shapes the smoothness of the failure curve, therefore ,we assign the effective temperature to a measure of soliton state in <k>-t curves. This inference suggests that soliton can be used equally as measure of effective temperature.


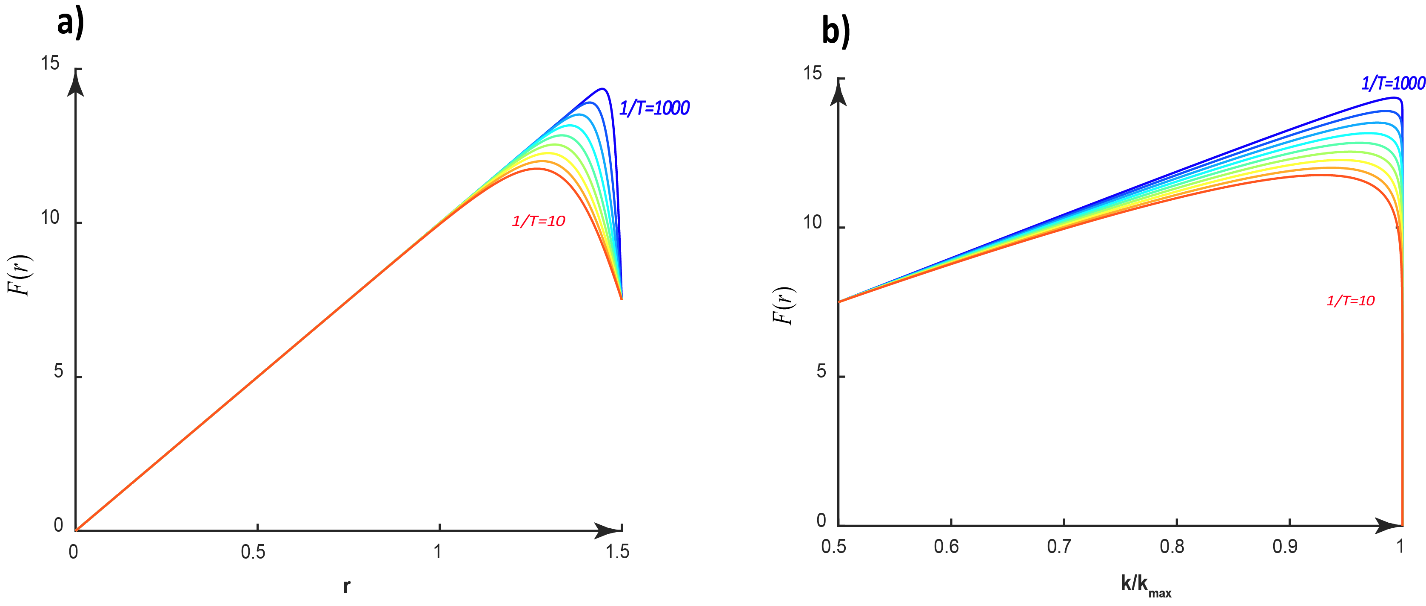


**Fig. S10|** **a)** cohesive force versus interatomic separation based on *Buehler-Gao’s model* for different temperatures. Approaching T→0 , the failure curve is more abrupt. **b)** Variation of interatomic separation versus a state of a site (*k_i_*) and for different *k_B_T* with *k_B_=1.* We have used $r_{i}=r_{c}+k_{B}T\log(\frac{\sum_{m} k_{i}}{k_{i}}-1)$with $r_{c}=1.5$ is the maximum allowed separation of the bonds (i.e., energy barrier in terms of thermal activation) and *m* is the number of energy-levels (communities in the k-chain).

***References***:

[1] Thompson, B. D., Young, R. P. & Lockner, D. A. Premonitory acoustic emissions and stick-slip in natural and smooth-faulted Westerly granite. *J Geophys Res*. 114, B02205J (2009).

[2] Ghaffari, H. O., Nasseri, M. H. B. & Young, R. P. Faulting of Rocks in a Three-Dimensional Stress Field by Micro-Anticracks. *Scientific Report*, 4 (2014).

[3] Buehler, M.J. and Gao, H., 2006. Dynamical fracture instabilities due to local hyperelasticity at crack tips. Nature, 439(7074), p.307.

[4] Sethna, J. P. *Statistical Mechanics: Entropy, Order Parameters and Complexity* (Oxford Univ. Press, 2006).
